# A Novel Grayscale Image Encryption Scheme Based on the Block-Level Swapping of Pixels and the Chaotic System

**DOI:** 10.3390/s22166243

**Published:** 2022-08-19

**Authors:** Muhammad Hanif, Nadeem Iqbal, Fida Ur Rahman, Muhammad Adnan Khan, Taher M. Ghazal, Sagheer Abbas, Munir Ahmad, Hussam Al Hamadi, Chan Yeob Yeun

**Affiliations:** 1 Riphah Institute of Informatics, Riphah International University, Malakand Campus, Islamabad 46000, Pakistan; 2 Department of Computer Science and IT, University of Lahore, Lahore 54590, Pakistan; 3 Department of Computer Science and IT, University of Malakand, Chakdara 18800, Pakistan; 4 Department of Software, Gachon University, Seongnam 13120, Korea; 5 College of Computer and Information Technology, American University in the Emirates, Dubai Academic City, Dubai 503000, United Arab Emirates; 6 Center for Cyber Security, Faculty of Information Science and Technology, Universiti Kebangsaan Malaysia (UKM), Bangi 43600, Malaysia; 7 School of Computer Science, National College of Business Administration and Economics, Lahore 54000, Pakistan; 8 College of Engineering and IT, University of Dubai, Dubai 14143, United Arab Emirates; 9 Center for Cyber Physical Systems, Khalifa University, Abu Dhabi 127788, United Arab Emirates

**Keywords:** cryptography, encryption, image processing, cipher, information security

## Abstract

Hundreds of image encryption schemes have been conducted (as the literature review indicates). The majority of these schemes use pixels as building blocks for confusion and diffusion operations. Pixel-level operations are time-consuming and, thus, not suitable for many critical applications (e.g., telesurgery). Security is of the utmost importance while writing these schemes. This study aimed to provide a scheme based on block-level scrambling (with increased speed). Three streams of chaotic data were obtained through the intertwining logistic map (ILM). For a given image, the algorithm creates blocks of eight pixels. Two blocks (randomly selected from the long array of blocks) are swapped an arbitrary number of times. Two streams of random numbers facilitate this process. The scrambled image is further XORed with the key image generated through the third stream of random numbers to obtain the final cipher image. Plaintext sensitivity is incorporated through SHA-256 hash codes for the given image. The suggested cipher is subjected to a comprehensive set of security parameters, such as the key space, histogram, correlation coefficient, information entropy, differential attack, peak signal to noise ratio (PSNR), noise, and data loss attack, time complexity, and encryption throughput. In particular, the computational time of 0.1842 s and the throughput of 3.3488 Mbps of this scheme outperforms many published works, which bears immense promise for its real-world application.

## 1. Introduction

Different hardware and software products are changing the way we live. From telecommunications to natural language processing, from cloud server storage to artificial intelligence software, from robotics to varied computer vision applications—one can see the tremendous influences these products have on humanity. Moreover, digital cameras are all around us, which means pictures are being taken around the clock. Images are ubiquitous, e.g., in the form of selfies, family pictures, party pictures, pictures of different dignitaries, etc. Among these images, some are sensitive, e.g., images of spies in military and espionage settings or images of new products made by multinational companies. Storing these images on gadgets and transmitting them through public networks are risky since hackers seek such activities. Hence, appropriate steps must be taken to safeguard these images. One traditional way is via encryption, in which plaintext is scrambled and diffused to convert it into some unrecognizable and cloudy format. For this purpose, some historical ciphers built by cryptographers are RSA, AES, and DES. Unfortunately, they work on text data only; digital images consist of pixels [1], which are tiny pieces of information that have intensity values in the range of 0 to 255. These pixels have strong correlations with each other and are highly redundant in character. Thus, new mechanisms are required for image encryptions that use these image characteristics.

In the literature, many image encryption schemes exist for grayscale [1,2,3,4,5,6,7,8,9,10] and color images [11,12,13,14,15,16,17,18,19]. Most of these encryption algorithms are based on chaotic maps. These systems/maps produce random data, which are utilized in encryption processes, including transposition (scrambling or confusion) and substitution (diffusion). These maps have the characteristics of randomness, unpredictability, ergodicity, aperiodicity, mixing, etc. They are fully dependent on their primary conditions and system parameters. Thus, they produce different streams of random numbers upon minute changes in their preconditions and parameters. This is why researchers have used them widely in designing their image encryption schemes [20,21,22,23,24,25]. In this study, the intertwining logistic chaotic map (ILM) is used to fulfill the requirements of chaoticity in the transposition and substitution processes. The ILM produces three chaotic streams and is much more unpredictable in nature [26].

As described earlier, the basic building blocks of encryption schemes are transposition and substitution. Upon surveying the image encryption literature—image encryption schemes were cracked because of the different vulnerabilities and weaknesses presented in their designs. The vulnerabilities were weak points that were ignored by cryptographers while designing their schemes. Few encryption schemes among them were based only on the transposition mechanism [27,28,29]. These mechanisms could not withstand the security attacks, such as known- and chosen plaintext attacks. In these encryption schemes, pixels are only shuffled in specific ways, which are usually quite predictable since each time the same procedure is adopted. Thus the attacker easily guesses the pattern and breaks the cipher [27]. To safeguard against these limitations, the new scheme for image encryption comprises both operations of transposition and substitution. After incorporating both of these operations, some image encryption schemes [30,31,32] were also broken through with different attacks [33,34,35], such as the differential attack, chosen cipher attack, and chosen plaintext attack, respectively. The main reason behind the breakages/weaknesses was that the chaotic data rendered through chaotic systems utilized in the schemes were not connected to the plain image. Thus, each time, the same data were generated, no matter which input image was used. The attacker figured out this pattern, which led to the breakage of the cipher. Plaintext sensitivity is a remedy to this attack [36,37,38].

Besides the concern of security, time efficiency was an important factor for researchers to deliver speedy products. In this regard, they resorted to different approaches, such as swapping mechanisms, circular shift operations, cellular automaton, block mechanisms, and so on. Many image encryption schemes were developed upon the notion of blocks also being produced [39,40,41,42,43,44,45,46,47,48]. In [39], the scheme for the encryption of images was presented based on the block-based transformation algorithm. The pixels were grouped into blocks. After that, scrambling operations were carried out by rotating them in the right direction using the XOR operation. Yet another image encryption using block cipher was introduced by [40]. The authors used chaotic sequences and adequate modes of operation, such as the counter mode and cipher block chaining. Their model was inspired by the Rijndael cipher. The security analysis showed that their algorithm was robust and had good security effects. The image encryption algorithm based on the theory of quantum was presented in [41]. In particular, their scheme was based on the quantum Arnold transformation (QAT) and the sine cation model (SCM). In this work, the image’s gray pixel position information and the values of the image blocks were knitted into two qubit sequences. For the scrambling operation, the QAT was applied over the image blocks and the XOR operation was made over the scrambled image. Using the enhanced-logistic map (ELM) and modified zigzag transformation, another block-based image encryption was proposed in [42]. The results demonstrated that their scheme could resist the various attacks of cryptanalysis.

Considering the literature review (as mentioned above), a new block image encryption scheme has been suggested, with the following highlights.

- The scheme is efficient regarding the computational time. Thus, it has good chances for its real-world application.
- This scheme has achieved better throughput. Moreover, the incorporation of plaintext sensitivity is a good way to avert the potential threats of cryptanalytic attacks.
- The majority of instructions of the suggested scheme are repetitive. Thus this scheme can be easily customized to run in some parallel settings.

The remainder of this paper is divided into five sections. In Section 2, the basic principles of block-level swapping and the chaotic system are discussed. The mechanisms for key stream generation and encryption/decryption procedures are discussed in Section 3. The simulation, security, and performance analyses using the varied validation metrics are presented in Section 4 and Section 5. Finally, the concluding remarks are provided in Section 6.

## 2. Basic Principles

The basic working principles upon which the current work depends are discussed in this section.

### 2.1. Chaotic System

The chaos theory probes into the systems that are highly dynamical in their characters and orientations. Moreover, they are extremely sensitive to the two components. These are the system parameters and the initial values of the chaotic system/map. In this work, the intertwining logistic map (ILM) has been used [49].

(1) la+1=μ×k1×mn×1−la+namod 1 ma+1=μ×k2×ma+na×11+la+12mod 1na+1=μ×la+1+ma+1+k3×sinnamod 1

**Figure 1:**
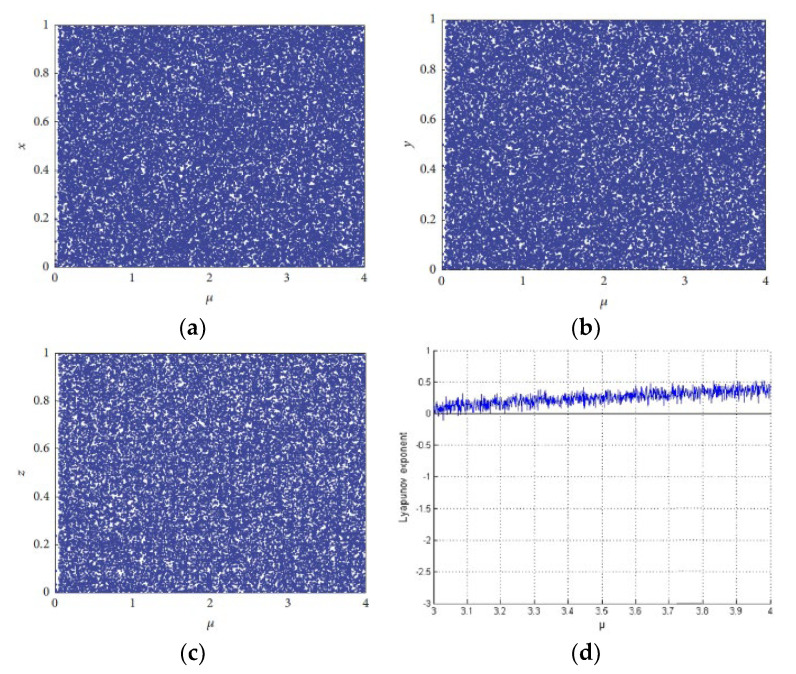
Distribution of intertwining logistic map; (**a**) bifurcation diagram of sequences x; (**b**) bifurcation diagram of sequences y; (**c**) bifurcation diagram of sequences z; (**d**) Lyapunov exponents diagram.

In the above equation, 0 < *μ* ≤ 3.999, |*k*_1_| > 33.500, |*k*_2_| > 37.970, and |*k*_3_| > 35.700 are the initial values. The ILM produces three chaotic streams, $\left(l,m,n\right)$ as can be seen in the above equation. This map is better than its antecedent logistic map since it has better chaoticity and contains no blank spaces [49]. This map has a desirable feature of chaoticity as this surpasses its predecessor maps. Additionally, there are no empty values and it has an even distribution as depicted in Figure 1a–c. Moreover, it has positive Lyapunov exponents, as shown in Figure 1d.

### 2.2. Block Swapping

**Figure 2:**
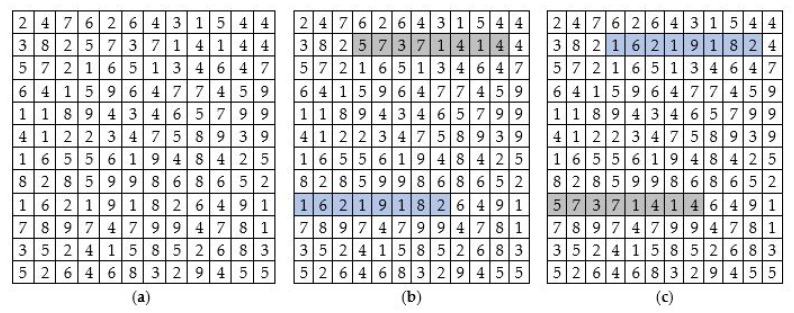
Block-swapping row-wise: (**a**) the initial state of the image; (**b**) the highlighted blocks; (**c**) the state of the image in (**a**) after block-swapping.

Blocks are fixed-size groups of pixels treated as one unit. They can help in improving the computational time of the cipher. In image encryption technology, a pixel is usually treated as a single unit, which is time-consuming. In contrast to that, the proposed scheme has treated 8 pixels as a single unit, dubbed a block. Figure 2 shows the block-wise swapping of the 8 pixels.

## 3. Proposed Block-Based Image Encryption Scheme

In this section, we discuss how the chaotic data were generated as well as the suggested scheme for the encryption of images.

### 3.1. Generation of the Initial Values and System Parameters

Two different types of keys are used in this scheme: a 256-bit hash code generated from the input image and a 256-bit user key given by the user. The hash key of the plain image helps in the realization of the plain text sensitivity. The hash key and the user key were mixed to generate the system parameters and the initial values of the chaotic map. Both the hash value and user key are split into four blocks of 64 bits each. The 256-bit hash value *HV* and User key *UK* are stated as follows:

(2) $$HV=hv_{1},hv_{2},hv_{3},hv_{4}.$$

(3) $$UK=uk_{1},uk_{2},uk_{3},uk_{4}.$$

subject to $hv_{a}=\left\{hv_{a,0},hv_{a,1},\ldots ,hv_{a,63}\right\},$ where in *hv*_*a*,*b*_, *a* denotes the character number and *b* denotes the bit number in *hv*_*a*,*b*_. Analogously, in the User key *UK*: $uk_{a}=\left\{uk_{a,0},uk_{a,1},\ldots ,uk_{a,63}\right\}$, where in *uk*_*a*,*b*_, *a* denotes the character number and *b* denotes the bit number in *uk*_*a*,*b*_. The following steps show the initial value and key stream generations for the ILM.

**Step 1:** Both the *HV* and *UK* are reshaped into 4 × 64 tables.

**Step 2:** The XOR operation is made between HV and UK, starting from the first row of the first table and the last row of the second table, as described by the following equations.

(4) $$R_{1,b}{}^{\prime }=HV_{1,b}⊕UK_{4,b}$$

(5) $$R_{2,b}{}^{\prime }=HV_{2,b}⊕UK_{3,b}$$

(6) $$R_{3,b}{}^{\prime }=HV_{3,b}⊕UK_{2,b}$$

(7) $$R_{4,b}{}^{\prime }=HV_{4,b}⊕UK_{1,b}$$

(8) $$R^{\prime }=R_{1,b},R_{2,b},R_{3,b},R_{4,b}$$

where the symbol ⊕ represents the XOR operation. Moreover, $R_{1,b}{}^{\prime }$ is the first row of the key table obtained after an XOR operation between $HV_{1,b}$ and $UK_{4,b}$. Similarly, other rows have been treated. Lastly, $R^{\prime }$ is the new 256-bit key value.

**Step 3:** After adding the values of columns for all four rows, we obtain the following:

(9) $$\alpha =\sum_{b=1}^{8}R^{\prime }\left(1,b\right)$$

(10) $$\beta =\sum_{b=1}^{8}R^{\prime }\left(2,b\right)$$

(11) $$\gamma =\sum_{b=1}^{8}R^{\prime }\left(3,b\right)$$

(12) $$\delta =\sum_{b=1}^{8}R^{\prime }\left(4,b\right)$$

**Step 4:** The equations below were used to calculate the ILM system parameters:

(13) $$k_{1}=\frac{\alpha ⊕\beta }{256}+33.50$$

(14) $$k_{2}=\frac{\gamma ⊕\delta }{256}+37.90$$

(15) $$k_{3}=\frac{\left(\alpha +\beta \right)⊕\left(\gamma +\delta \right)}{256}+35.70$$

(16) $$\mu =mod\left(\left(\alpha +\left(\gamma /\delta \right)\times \beta \right),\;4\right)$$

**Step 5:** The initial values of the ILM were calculated as

(17) $$l_{0}=mod\left(\left(\mu \times \left(k_{1}+k_{2}\right)/k_{3}\right),\;0.5\right)$$

(18) $$m_{0}=mod\left(\left(\mu \times \left(k_{2}+k_{3}\right)/k_{1}\right),\;0.5\right)$$

(19) $$n_{0}=mod\left(\left(\mu \times \left(k_{1}+k_{3}\right)/k_{2}\right),2.5\right)$$

where *mod*(*y*, *z*) calculates the remainder when *z* divides the *y*.

**Step 6:** The chaotic system (1) is repeated for ($MN+n_{0}$) times to obtain three chaotic steams, i.e., *l*, *m*, *n*, where the $l=\left[l_{1},l_{2},\ldots ,\;l_{MN+n0}\right]$, *m* $=\left[m_{1},m_{2},\ldots ,\;m_{MN+n0}\right]$, $n=\left[n_{1},n_{2},\ldots ,\;n_{MN+n0}\right]$. Here, MN are the resolutions of input images and *n*_0_ ≥ 500 are used for the removal of momentary effects from the chaotic map by ignoring the starting *n*_0_ values.

**Step 7:** The three chaotic streams of ILM i.e., *l*, *m*, and *n* are further modified as follows.

(20) $$block-selection1\left(i\right)=mod\left(floor\left(l\left(i\right)\times 10^{14}\right),NoB\right)$$

(21) $$block-selection2\left(i\right)=mod\left(floor\left(m\left(i\right)\times 10^{14}\right),NoB\right)$$

(22) $$key-image\left(i\right)=mod\left(floor\left(n\left(i\right)\times 10^{14}\right),256\right)$$

where NoB denotes the number of blocks. Further, block−selection1, block−selection2, and key−image are the new key streams according to the algorithmic logic we conceived. *i* = 1, 2, …, *MN*.

### 3.2. Encryption Procedure

**Figure 3:**
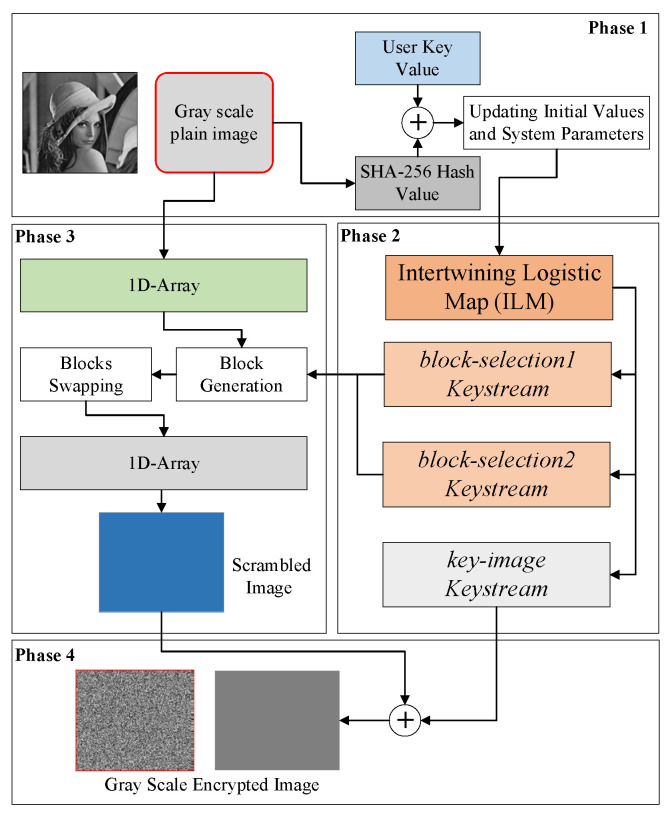
Block-based encryption scheme.

The proposed image encryption scheme is shown in Figure 3. The encryption procedure is explained in the steps below.

**Step 1:** This involves inputting the grayscale image and decomposing it into the 1D array. The grayscale plain image *img* of size M×N is input. The input image is then decomposed into the one-dimension (1D) array, i.e., *Array*. The size of this 1D array is 1×M×N.

**Step 2:** This involves decomposing the 1D array into blocks. Decompose the 1D array into blocks; each block size is 64 bits or 8 pixels. The total number of blocks is *NoB*, obtained as follows.

(23) $$NoB=\left(1\times M\times N\right)/8$$

**Step 3:** Scrambling operation.

**Step 3.1:** Set the *index* = 1.

**Step 3.2:** Block selection. Select the first and second blocks and assign them to *bs1* and *bs2*, as follows.

(24) $$bs1=block-selection1\left(index\right)\times 8+1$$

(25) $$bs2=block-selection2\left(index\right)\times 8+1$$

**Step 3.3:** Swapping operation.

The following steps were carried out to perform the swapping operations over the selected blocks.

(26) $$emp=Array\left(bs1:bs1+8\right)$$

(27) $$Array\left(bs1:bs1+8\right)=Array\left(bs2:bs2+8\right)$$

(28) $$Array\left(bs2:bs2+8\right)=temp$$

Here, the variable Temp was used to store the block of the pixels.

**Step 3.4:***index* = *index* + 1.

**Step 3.5:** Repeat Steps 4.2, 4.3, and 4.4, while index≤MN.

**Step 3.6:** Let $Array^{\prime }=Array$.

*Array’* is the scrambled image.

**Step 4:** Diffusion operation.

Diffusion effects were realized through the XOR operation between $Array^{\prime }$ and the key−image.

(29) $$Array^{\prime \prime }\left(a\right)=Array^{\prime }\left(a\right)⊕key-image\left(a\right)$$

where *a* = 1, 2, …, *MN*. Reshape the image $Array^{\prime \prime }$ to *M* × *N* to obtain the final cipher image.

In the domain of cryptography, two approaches exist for the task of encryption, i.e., the private key and the public key. In this work, we adopted the former approach. Thus, the decryption procedure does not need to be explained in detail. This procedure would just be a reversal of the steps of the encryption procedure.

## 4. Simulation

**Figure 4:**
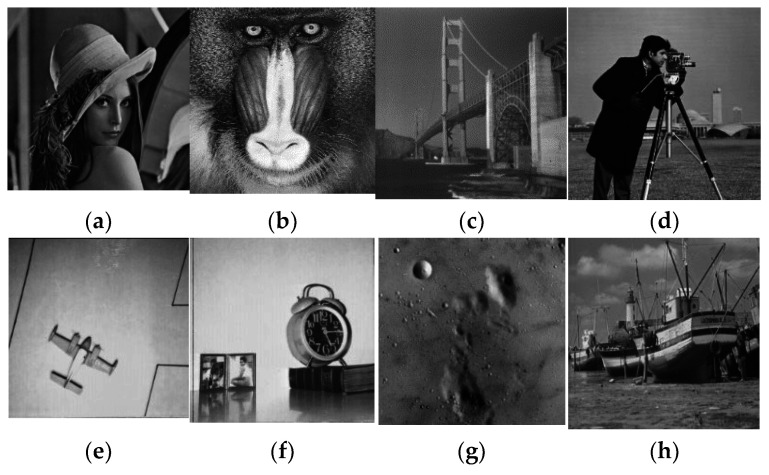
Original input images: (**a**) Lena; (**b**) baboon; (**c**) bridge; (**d**) cameraman; (**e**) airplane; (**f**) clock; (**g**) moon; (**h**) ship.

**Figure 5:**
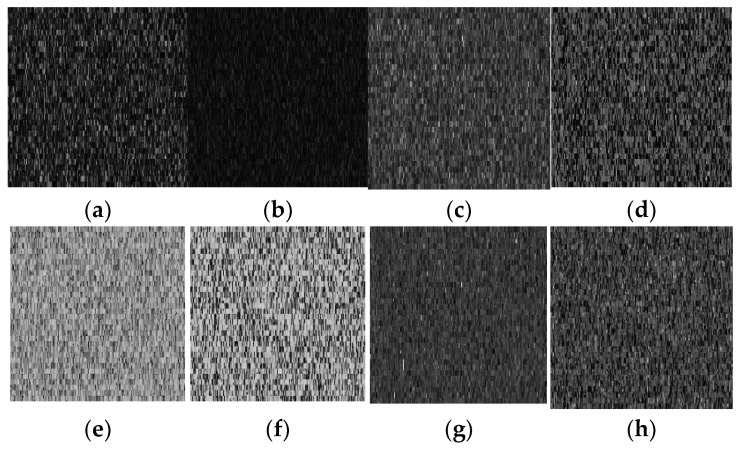
Scrambled images: (**a**) Lena; (**b**) baboon; (**c**) bridge; (**d**) cameraman; (**e**) airplane; (**f**) clock; (**g**) moon; (**h**) ship.

**Figure 6:**
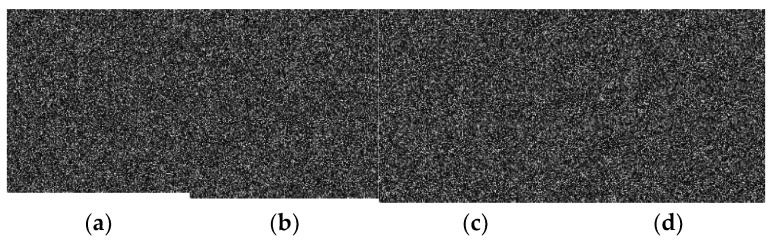

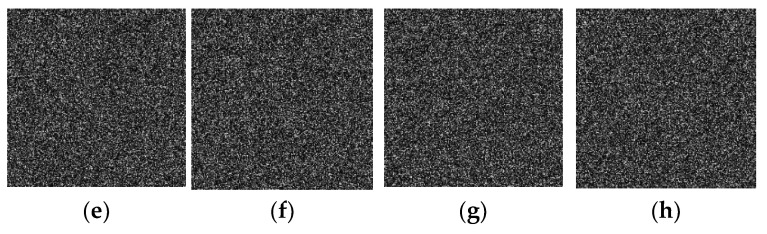
Encrypted images: (**a**) Lena; (**b**) baboon; (**c**) bridge; (**d**) cameraman; (**e**) airplane; (**f**) clock; (**g**) moon; (**h**) ship.

**Figure 7:**
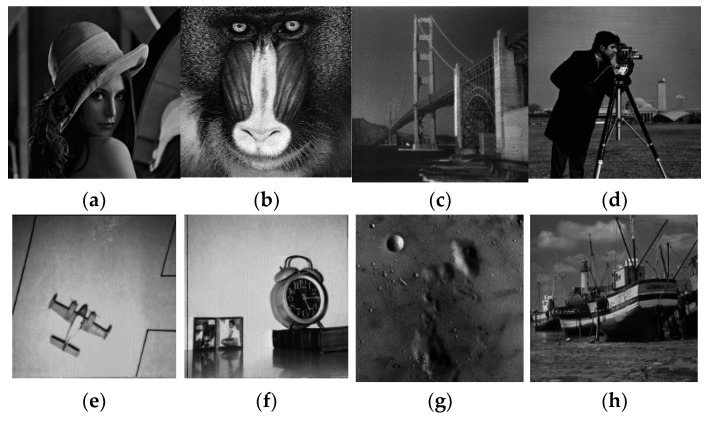
Decrypted images: (**a**) Lena; (**b**) baboon; (**c**) bridge; (**d**) cameraman; (**e**) airplane; (**f**) clock; (**g**) moon; (**h**) ship.

A good cipher must be capable of handling a variety of attacks launched by potential antagonists. The differential attacks, chosen plaintext attack, brute force attack, entropy attack, cipher attack, statistical attack, and many others, are common in the realm of image encryption. To demonstrate it, eight grayscale images were chosen, each with a size of 256 × 256. The grayscale images were downloaded from the online repository of images using the link: http://sipi.usc.edu/database/ (accessed on 4 December 2021). The selected grayscale images were: Lena, baboon, bridge, cameraman, airplane, clock, moon, and ship. MATLAB version R2018a (64-bits), double-precision, was used (according to the IEEE [50] standard 754). The variable values used in the ILM were: *k*_1_ = 33.5, *k*_2_ = 37.9, *k*_3_ = 35.7, *x*_0_ = 0, *y*_0_ = 0, *z*_0_ = 0, μ=0. Figure 4, Figure 5, Figure 6 and Figure 7 show the original plain (input) images, scrambled images, encrypted images, and decrypted images, respectively. These figures clearly show that the inputted plain images were converted into unrecognizable formats. The attacker would have no clue on how to retrieve the original input images from the scrambled and output encrypted images.

## 5. Security Analysis

In this section, the performance and security analyses based on different validation metrics are carried out.

### 5.1. Key Space Analysis

In any encryption scheme, one of the most important features is the key space. A large key space provides resistance against a brute force attack. There are four blocks. Each block consists of 64 bits, contributing ${\left(2^{64}\right)}^{4}=2^{256}$ to the key space. Further, the ILM has four system parameters and three initial values making up seven variables. Moreover, $10^{-15}$ is taken as the computer precision. Thus, this contributes ${\left(10^{15}\right)}^{7}=10^{105}$ to the key space. Therefore, the overall key space comes out as $2^{256}\times 10^{105}=1.16\times 10^{182}$. This value is sufficient to counter the brute force threat since it crosses the minimum threshold $2^{100}$ [17,22]. Table 1 highlights the key space of our proposed scheme and its comparison with other published works.

### 5.2. Statistical Analysis

In image encryption technology, another significant metric is the statistical analysis. Two types of tests have been conducted by researchers, i.e., the histogram analysis and correlation coefficient analysis.

#### 5.2.1. Histogram Analysis

**Figure 8:**
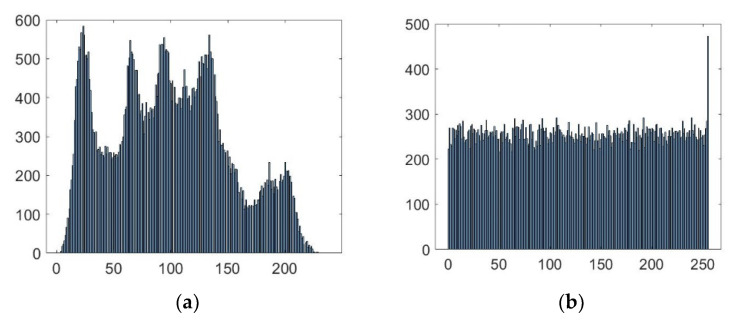
The Lena grayscale image histogram: (**a**) plain image; (**b**) encrypted image.

In a given image, the pixel intensity value distribution is provided through the histogram. For a plain image, the histogram has slanting bars, which can be exploited by a hacker to obtain useful information about the image. To resist the statistical attack, a cipher must be capable of converting the slanting bars into a well-organized plain bar with almost the same distribution. In this way, a hacker would not be able to obtain any useful information. The histograms of both plain and cipher images of Lena are shown in Figure 8. Figure 8a shows that the histogram of the Lena plain image has curved slanting bars. In contrast, Figure 8b shows that the histogram is a well-organized plain bar with uniform distribution. These well-organized plain bars provide great immunity against the histogram attack. This shows that the proposed scheme is efficient.

#### 5.2.2. Analysis of the Correlation Coefficient

For any plain and natural images, the pixels are arranged in systematic ways. The close pixels are correlated in an intense manner. The correlation coefficient (*CC*) is another security parameter by which the inter-pixel correlation is found. These adjacent pixels are diagonally, vertically, or horizontally aligned to one another. Image ciphers are expected to disrupt these adjacent pixels. To analyze the *CC* of the proposed scheme, we took 3000 pairs of consecutive pixels from both the cipher and original images in an arbitrary way. *CC* was calculated using the following equation:

(30) $$CC=\frac{A\sum_{l=1}^{A}\left(x_{l}\times y_{l}\right)-\sum_{l=1}^{A}x_{l}\times \sum_{l=1}^{A}y_{l}}{\sqrt{\left(A\sum_{l=1}^{A}x_{l}^{2}-\left(\sum_{l=1}^{A}x_{l}{}^{2}\right)\right)\left(A\sum_{l=1}^{A}y_{l}^{2}-\left(\sum_{l=1}^{A}y_{l}{}^{2}\right)\right)}}$$

**Figure 9:**
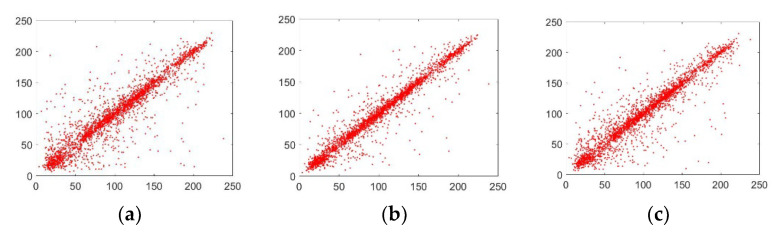

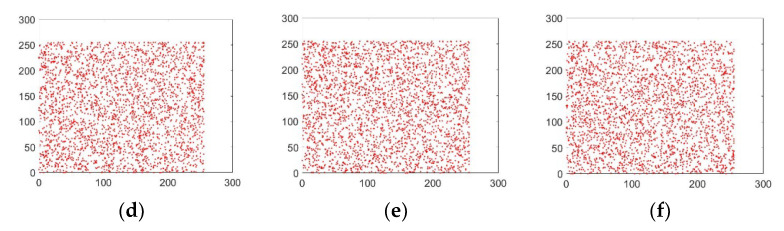
The adjacent pixel correlation distribution with directions: (**a**) horizontal component of the input Lena plain image; (**b**) vertical component of the input Lena plain image; (**c**) diagonal component of the input Lena plain image; (**d**) horizontal component of the generated Lena encrypted image; (**e**) vertical component of the generated Lena encrypted image; (**f**) diagonal component of the generated Lena encrypted image.

Here, *A* denotes the number of pixels; the neighboring pixels are referred to by *x* and *y*. The correlation distribution for the adjacent pixels is shown in Figure 9.

Figure 9 demonstrates that the cipher and plain images are extremely distinct from each other, asserting the success of the suggested image cipher.

### 5.3. Analysis of Information Entropy

The metric of information entropy (IE) could be used to judge the randomness and arbitrariness in some images. Shannon [55] in 1949 provided the concept of IE using the following mathematical equation:

(31) $$E\left(k\right)=\sum_{i=0}^{2^{n}-1}d\left(k_{i}\right)log\frac{1}{d\left(k_{i}\right)}$$

where $E\left(k\right)$ is the IE of the information source k. The probability of $k_{i}$ is represented by $d\left(k_{i}\right)$, and the number of the given image pixels is represented by n. Moreover, the largest value of this metric is calculated as 8 for any encrypted image with 256 grayscale values.

Table 2 demonstrate that the cipher and plain images are extremely distinct from each other, asserting the success of the suggested image cipher. It also observed that the cipher and plain images are extremely distinct from each other, asserting the success of the suggested image cipher.

The results of IE of our proposed scheme are presented in Table 3. The calculated average IE for the encrypted images is 7.9955, which is near 8.

### 5.4. Plaintext Sensitivity Analysis (Differential Attack)

Cryptanalysts exhaust all possibilities to hack the hidden key of a security product. In this vast range of attacks, the differential attack is included. In the special attack dynamics, the cryptanalyst encrypts a plain image and obtains its encrypted version. Further, one more encrypted image is obtained after making a tiny alteration in the same input image by changing a single pixel value. The discovery of the confidential key can potentially be achieved by closely inspecting these two cipher images. In the literature, two validation metrics were employed to investigate the prowess and immunity of an encryption scheme for the images against differential attacks. These were the unified average changing intensity (*UACI*) and the ‘number of pixels change rate’ (*NPCR*). The following mathematical equations are used to find these two metrics.

(32) $$NPCR=\frac{\sum_{a,b}D\left(a.b\right)}{C\times D}\times 100\%$$

Here, the dimensions of the images are denoted by C and D. Further, D(a, b) is defined as:

(33) $$D\left(a,b\right)=\left\{\begin{array}{c} 1,if\;C\left(a,b\right)\neq C^{\prime }\left(a,b\right),\\ 0,if\;C\left(a,b\right)=C^{\prime }\left(a,b\right) \end{array}\right.$$

(34) $$UACI=\frac{1}{C\times D}\left[\sum_{i,j}\frac{\left|C\left(a,b\right)-C^{\prime }\left(a,b\right)\right|}{255}\right]\times 100\%$$

In these equations, C and C′ denote the encrypted images with a change in the pixel value and no change in the pixel value, respectively.

Table 4 shows the average values of NPCR and UACI for the chosen eight images, i.e., 99.6282 and 33.2459, respectively.

A comparison has also been made in Table 5. Additionally, Table 5 shows *CC* results between neighboring pixels for the input Lena plain image and its encrypted version. It is clear from Table 5 that the results approximate to one for the plain image and zero for the cipher image. Moreover, Table 5 presents a comparison of this security parameter between the published works and the proposed scheme. One can see that the results are comparable. The NPCR results of the proposed scheme are better than the ones in [40,45,51,52,53,54]. Moreover, the proposed cipher could only beat [53] regarding UACI.

### 5.5. Peak Signal-to-Noise Ratio (PSNR) Analysis

The basic aim of any image encryption scheme is to cause a maximum difference between the plain image and its encrypted version. This metric is employed for this purpose by the cryptographers whose mathematical formula is

(35) PSNR=20 log10255MSEdBMSE=1A×B∑k=1A∑l=1BP0k,l−P1k,l2

where *A* and *B* are the dimensions of the image. *P*_0_(*k*, *l*) and *P*_1_(*k*, *l*) refer to the intensity values of pixels of plain and cipher images. The mean squared error (*MSE*) is the error between the two images. *PSNR* and *MSE* are inversely proportional to each other, as the equation implies. The higher the *MSE* value, the better the scheme will be. Analogously, a lower value of *PSNR* is desirable.

Table 6 shows the *PSNR* values for the plain, cipher, and decrypted images. The first row of this table has the entries of infinity (Inf) for (O–D). This indicates that plain and decrypted images are exactly the same. Further, this occurred due to the factor MSE=0. This further implies that the proposed scheme is lossless. Moreover, the second row of Table 6 shows the values for (O–C), which are better than the ones given in [58,59,60]. These stats depict that the proposed scheme is better. A comparative analysis between the published works and the suggested scheme can be seen in Table 6. The IE of the Lena image and the mean values of all the chosen images of the suggested scheme are superior to the one in [39]. Hence, the suggested image cipher is immune to the entropy attack.

### 5.6. Noise and Data Loss Analysis

**Figure 10:**
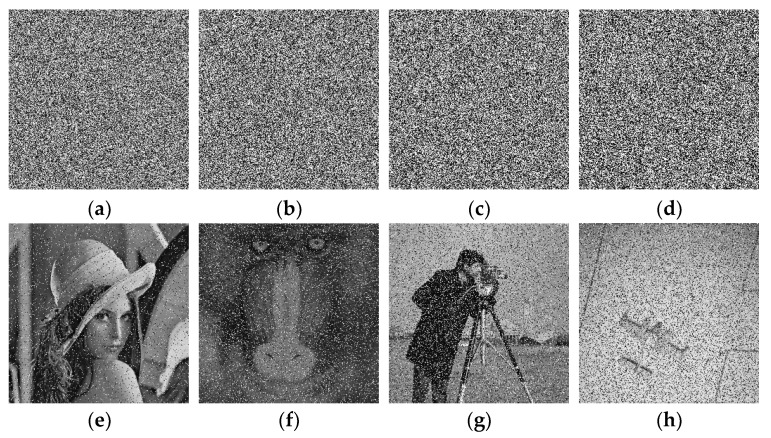
Pepper and salt noise attacks with different densities: (**a**) Lena cipher image by accumulating pepper and salt noise with noise density 0.1; (**b**) baboon cipher image by accumulating pepper and salt noise with noise density 0.2; (**c**) cameraman cipher image by accumulating pepper and salt noise with noise density 0.3; (**d**) airplane cipher image by accumulating pepper and salt noise with noise density 0.4; (**e**) the decrypted image obtained from (**a**); (**f**) the decrypted image obtained from (**b**); (**g**) the decrypted image obtained from (**c**); and (**h**) the decrypted image obtained from (**d**).

**Figure 11:**
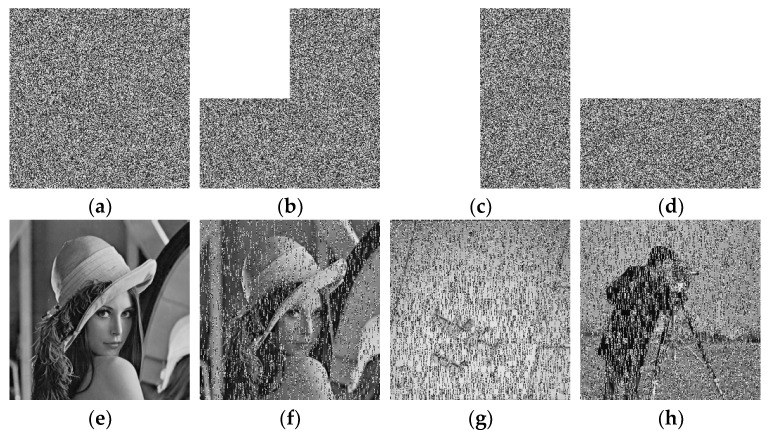
Analysis of the data loss attack: (**a**) 0% data loss in the encrypted image of Lena; (**b**) 25% data loss in the encrypted image of Lena; (**c**) 50% data loss in the encrypted image of the airplane; (**d**) 50% data loss in the encrypted cameraman image; (**e**) decrypted Lena image from the image drawn in (**a**); (**f**) decrypted Lena image from the image drawn in (**b**); (**g**) decrypted airplane image from the image drawn in (**c**); (**h**) decrypted cameraman image from the image drawn in (**d**).

In a real-time scenario, the images are vulnerable to the assaults of data loss and noise. A good scheme is expected to successfully cope with them. Figure 10 shows the noise analysis. The pepper and salt noise was mixed with various densities of 0.1, 0.2, 0.3, and 0.4 in the cipher images of Lena, baboon, cameraman, and airplane (Figure 10a–d). The images restored after applying the decryption algorithm over them were redrawn in Figure 10e–h, respectively. Obviously, these decrypted images are still recognizable, which demonstrates that the suggested scheme can avert the noise attack. Similarly, a data loss analysis is demonstrated in Figure 11. Figure 11a–d represent the encrypted images with 0%, 25%, 50%, and 50% data losses in the encrypted images of Lena, Lena, airplane, and cameraman, respectively. The decrypted images are shown in Figure 11e–h. One can see that the plain images can be appreciated easily, implying that the proposed image cipher has the capability to foil data loss attacks.

### 5.7. Computational Time Analysis

Apart from security concerns, speedy ciphers are more demanding in this modern world. The proposed work was carried out using the system with the specification of Intel^®^ Core™ i7-3740QM CUP@2.70 GHz, 8GB RAM. Further, the Windows 10 Education version operating system was used with MATLAB R2018a.

Table 7 shows the execution times for the encryption and decryption algorithms against the chosen images. Upon calculating the average values for encryption and decryption algorithms of the chosen images, we obtained 0.1830 and 0.1831 s. Moreover, the execution time was far better than the published works [40,61] because this scheme is based on the block-level swapping of pixels, due to which we gained a dramatic increase in computational time.

Apart from this, there is another associated concept called encryption throughput (*ET*). This refers to the dimensions of the image is encrypted/decrypted in some unit time. Its equation is

(36) $$ET=\frac{Image_{Size}\left(Bite\right)}{Encryption_{Time}\left(Second\right)}$$

Table 8 demonstrates the encryption throughout the suggested scheme along with its comparison with other works. The results show that the proposed scheme vividly outperforms these works regarding the important metric of the *ET*.

The time complexity calculation of the proposed algorithm is as follows. Step 6 of Section 3.1 takes O(3MN) to generate the three streams of random numbers of the chaotic map employed in this work. Now, we work on Section 3.2. Step 4.2 contributes O(2MN) to the complexity. Further, the time complexity for the swapping operations takes O(3MN). The simple assignment operation of Step 4.4 takes O(MN). Lastly, Step 5 for the XOR operation consumes O(MN). By adding all of these time complexities, we obtain O(10 MN) as the time complexity for the suggested cipher. This time complexity beats these studies [11,16,63], since the computational complexities of these studies are O(15 MN + $24\sqrt{\backslash MN}$), O(24 MN), and O(24 MN), respectively. The proposed cipher beats [11,16,63] by a factor of $\frac{24MN}{10MN}=2.4\;\left(43\right)$.

(37) $$\frac{15MN+24\sqrt{MN}}{10MN}=\frac{15\times 256\times 256+24\sqrt{256\times 256}}{10\times 256\times 256}\;\approx 1.505$$

at *M* = 256 and *M* = 256. As the dimensions of the input images increase, this factor will also increase.

## 6. Conclusions

Upon surveying images from the literature cryptography, one will find that many schemes for image encryption have been written at different granularity levels. These include bits, pixels, DNA strands, and block-level. To expedite the speed, we proposed a new image encryption algorithm in this study. The underlying idea that differentiates it from the other works is that the whole block of pixels has been swapped with another block of pixels. This act gave competitive results as far as the speed and encryption throughput are concerned. The given image is reshaped into a linear array. Through the streams of random numbers, two blocks were selected, consisting of eight pixels. After selection, they were swapped with each other. This action was repeated for an arbitrary number of times. The scrambled image was further XORed with the last and third streams of chaotic data to obtain the final cipher image. The intertwining logistic map was employed in this work. The essential feature of plaintext sensitivity was realized by adding SHA-256 hash codes. An exhaustive security analysis and machine experiments vividly demonstrated the robust defiance of ubiquitous threats from the cryptanalysis community and the chances for real-world applications of the suggested image cipher. In particular, we gained encryption and decryption speeds of 0.1842 and 0.1861 s, respectively, which no doubt gave a major push to the state-of-the-art. In the future, we intend to inject the DNA strands to come up with more security and defiance to potential threats. Moreover, one limitation plagues the proposed cipher, i.e., the sides of the resolution of the given input image must be a multiple of 8. In the future, we will extend our work so that it may cater to all dimensions of the given images.

## Figures and Tables

**Table 1:** Key space comparison between the proposed scheme and other schemes.

Algorithm	Key Space
Ours	1.16 × 10^182^
[19]	10^105^
[45]	$2^{197}\approx 2\times 10^{59}$
[51]	10^128^
[52]	10^90^
[53]	$2^{197}\approx 2\times 10^{59}$
[54]	$2^{199}\approx 8\times 10^{59}$

**Table 2:** The comparison of the correlation coefficient(s) (*CC*) between our proposed image encryption scheme with other image encryption schemes.

Images	Encryption Algorithm	Correlation Direction		
		Horizontal	Vertical	Diagonal
Original Lena image	Our Algorithm	0.8941	0.9172	0.9516
Encrypted Lena image	Our Algorithm	0.0065	−0.0016	0.0063
Lena	[45]	−0.0164	−0.0083	0.0080
Lena	[51]	0.0038	0.0024	0.0052
Lena	[52]	0.0044	0.0151	0.0012
Lena	[53]	−0.0077	0.0117	0.0119
Peppers	[54]	0.0171	−0.0213	0.0118
MRI	[56]	0.0060	0.0123	0.0023
Lena	[57]	0.0038	−0.0011	0.0010

**Table 3:** The information entropy (IE) results analysis between our proposed image encryption scheme and other schemes.

Encryption Algorithm	Images	Size	Original	Encrypted
Our Algorithm	Lena	256 × 256	7.5690	7.9957
	Baboon	256 × 256	6.6962	7.9952
	Bridge	256 × 256	7.0097	7.9960
	Cameraman	256 × 256	6.4523	7.9952
	Airplane	256 × 256	6.2616	7.9954
	Clock	256 × 256	6.7057	7.9955
	Moon	256 × 256	7.1701	7.9956
	Ship	256 × 256	6.7093	7.9956
	**Average**	**256 × 256**	**6.8217**	**7.9955**
[40]	Lena	256 × 256	6.3872	7.9953
[45]	Lena	512 × 512	7.4456	7.9994
[51]	Lena	256 × 256	7.5683	7.9971
[52]	Lena	512 × 512	7.4456	7.9993
[53]	Lena	512 × 512	7.4456	7.9994

**Table 4:** The calculated average values of NPCR and UACI for different images.

Images	NPCR	UACI
Lena	99.5743	33.0509
Baboon	99.6521	33.1627
Bridge	99.6506	33.3766
Cameraman	99.6445	33.6619
Airplane	99.6518	33.8155
Clock	99.6323	33.1338
Moon	99.6216	32.5399
Ship	99.5987	33.2262
**Average**	**99.6282**	**33.2459**

**Table 5:** The calculated average values of NPCR and UACI of our proposed scheme and its results comparisons with other existing encryption schemes.

Algorithm	Average NPCR	Average UACI
Ours	99.6282	33.2459
[40]	99.6091	33.4437
[45]	99.6000	33.4000
[51]	99.6000	33.4000
[52]	99.6200	33.4500
[53]	99.6100	33.4200
[54]	99.6110	33.2320

**Table 6:** The PSNR results between the plain, cipher, and decrypted images: ‘O–C’ stands for the original and cipher images, ‘O–D’ stands for the original and decrypted images.

Encryption Algorithm	PSNR	Lena	Baboon	Bridge	Cameraman	Airplane	Clock	Moon	Ship
Our algorithm	PSNR(O–D)	Inf	Inf	Inf	Inf	Inf	Inf	Inf	Inf
	PSNR(O–C)	8.5534	8.0991	8.3854	7.7515	9.9684	7.2682	9.3150	9.1370
[58]	PSNR(O–D)	96.2956							
	PSNR(O–C)	9.0348							
[59]	PSNR(O–C)	8.6878							
[60]	PSNR(O–C)	9.0486							

**Table 7:** Execution time of encryption (Enc) and decryption (Dec) in seconds.

Images	Enc	Dec
Lena	0.1842	0.1861
Baboon	0.1817	0.1879
Bridge	0.1869	0.1793
Cameraman	0.1842	0.1844
Airplane	0.1834	0.1834
Clock	0.1856	0.1870
Moon	0.1796	0.1839
Ship	0.1786	0.1733
**Average**	**0.1830**	**0.1831**
[40]	4.0200	-
[61]	1.4800	-

**Table 8:** Suggested scheme’s encryption throughout and a comparative analysis with other works.

Images	ET in Mbits
Lena	3.3488
Baboon	3.5257
Bridge	2.7833
Cameraman	2.8507
Airplane	3.3411
Clock	2.8292
Moon	3.4437
Ship	2.9401
**Average**	**3.1328**
[16]	0.4240
[40]	0.1419
[62]	2.3861

## Data Availability

The simulation files/data used to support the findings of this study are available from the corresponding author upon request.
